# The Role of Praziquantel in the Prevention and Treatment of Fibrosis Associated with Schistosomiasis: A Review

**DOI:** 10.1155/2022/1413711

**Published:** 2022-10-21

**Authors:** Xuehua Niu, Tao Hu, Ye Hong, Xiaoyan Li, Yuzhou Shen

**Affiliations:** Department of Gastroenterology, Kunshan Third People's Hospital, Kunshan 215300, Jiangsu Province, China

## Abstract

Schistosomiasis remains a major global public health concern. Currently, the control of this neglected tropical disease still depends on chemotherapy to reduce the prevalence and intensity of the parasite infection. It has been widely accepted that praziquantel is highly effective against all species of *Schistosoma*, and this agent is virtually the only drug of choice for the treatment of human schistosomiasis. Mass drug administration (MDA) with praziquantel has been shown to be effective in greatly reducing the prevalence and morbidity due to schistosomiasis worldwide. In addition to antischistosomal activity, a large number of experiential and clinical evidence has demonstrated the action of praziquantel against fibrosis caused by *S. mansoni* and *S. japonicum* infections through decreasing the expression of fibrotic biomarkers such as *α*-smooth muscle actin (*α*-SMA), collagen, matrix metalloproteinase (MMP), and tissue inhibitor of metalloproteinase (TIMP), and inhibiting the expression of proinflammatory cytokines such as interleukin (IL)-6, tumor necrosis factor (TNF)-*α,* and transforming growth factor (TGF)-*β*, as well as chemokines, and similar antifibrotic activity was observed in mouse models of fibrosis induced by carbon tetrachloride (CCl4) and concanavalin A (Con-A). In this review, we discuss the role of praziquantel in the prevention and treatment of fibrosis associated with schistosomiasis and the possible mechanisms. We call for randomized, controlled clinical trials to evaluate the efficacy and safety of praziquantel in the treatment of schistosomiasis-induced hepatic fibrosis, and further studies to investigate the potential of praziquantel against fibrosis associated with alcohol consumption, viruses, and toxins seem justified.

## 1. Introduction

Schistosomiasis, a neglected tropical disease caused by the blood fluke of the genus *Schistosoma*, remains a global public health concern that ranks second to malaria among human parasitic diseases in terms of the number of people infected and at risk of infection [1]. Worldwide, it is estimated that over 140 million people are thought to have the disease, with a further 779 million at risk of infection [2]. Although the elimination of this tropical parasitic disease requires a multidisciplinary integrated approach [3–5], the control of schistosomiasis still relies on chemotherapy, which has been proven to be effective in reducing the prevalence and intensity of the parasitic infection [6–8].

Hepatosplenic schistosomiasis is characterized by typical pathological changes at chronic and advanced stages, including egg granulomas, fibrosis, and tissue damage within the liver and other host tissues [1]. Hepatic fibrosis, which is characterized by excessive deposition of collagen and other extracellular matrix (ECM) components resulting from granulomatous responses triggered by soluble egg antigens (SEA) secreted by parasite eggs, is the main pathological mechanism and the major lesion of hepatosplenic schistosomiasis [9]. Hepatic stellate cells (HSCs), hepatic macrophages, immune cells, cytokines (interleukin (IL)-13, transformation growth factor (TGF)-*β*1, interferon (IFN)-*γ*), and microRNAs (miRNAs) have been found to contribute to the pathogenesis of schistosomiasis-induced hepatic fibrosis [10–17]. Portal hypertension and ascites associated with hepatic fibrosis have been identified as the main causes of mortality among patients with chronic hepatosplenic schistosomiasis [18], and currently, there is no cure for hepatic fibrosis except liver transplantation [19]. However, liver transplantation suffers from problems of lack of donors, high costs, and use of immunosuppressive agents, which limits its clinical applications [20]. A search for novel treatments for hepatic fibrosis is therefore given a high priority.

Since praziquantel, a broad-spectrum schistosomicide, was developed in 1970 s [21], it has replaced other antischistosomal agents to become the only drug of choice for treatment of human schistosomiasis due to high efficacy, low toxicity, easy administration, and low cost [22–24]. The agent is found to be active against all species of *Schistosoma*, notably, adult stages of the parasite [25]. As a consequence, the introduction of praziquantel led to the global schistosomiasis control strategy shifting from disease control to morbidity control [26]. Mass drug administration (MDA) with praziquantel has been shown to be effective in reducing the prevalence and morbidity due to schistosomiasis [27–29]. Furthermore, an antifibrotic activity of praziquantel was reported in both animal models and patients infected with schistosomiasis [30,31]. This review article aims to discuss the role of praziquantel in the prevention and control of fibrosis associated with schistosomiasis and the possible mechanisms.

## 2. Literature Search Strategy

A joint search was performed in international and national electronic databases, including Web of Knowledge, PubMed, Scopus, Google Scholar, Wanfang Data (https://www.wanfangdata.com.cn/), CNKI (https://www.cnki.net) and VIP (https://qikan.cqvip.com/) using the terms “schistosomiasis,” “fibrosis,” and “praziquantel” to retrieve publications concerning the action of praziquantel against fibrosis associated with schistosomiasis during the period from January 1st, 1970 to December 31st, 2021. Inclusion criteria involved: (1) studies reporting the activity of praziquantel against fibrosis associated with schistosomiasis; and (2) animal studies or clinical reports. Publications that met the following exclusion criteria were: (1) review articles; or (2) the full text was unavailable.

### 2.1. Experimental Evidence

In a murine model of *S. mansoni*-induced hepatic fibrosis, administration of praziquantel at a dose of 250 mg/kg modestly diminished liver fibrosis as compared to untreated controls 10 weeks post-treatment and suppressed fibrosis and reduced liver collagen content to normal levels 20 weeks post-treatment [32]. In Swiss albino mice experimentally infected with *S. mansoni*, praziquantel treatment resulted in a reduction in total collagen content and a recovery of the type III (Col3)/I collagen (Col1) ratio to normal limits [33]. In Syrian golden hamsters infected with 100 *S. mansoni* cercariae each, a significant reduction in hepatic and splenic granulomas, fibrosis, and circulating cathodic antigen (CCA), and circulating anodic antigen (CAA) was seen following praziquantel treatment [34]. Following praziquantel administration, amelioration of hepatic granulomas and reduction of *Col1*, *Col3,* and *Col4* gene expression were observed 6 and 12 months post-treatment, and 71.4% resorption of hepatic fibrous tissues was found 12 months post-treatment in a CBA/J^k^ mouse model of *S. mansoni* infections [35]. In Swiss Webster outbred mice infected with *S. mansoni*, treatment with praziquantel resulted in elimination of egg granulomas or collagen fibrils, and reduced the expression of signal transducer and activator of transcription 1 (STAT1), STAT4, IFN-*γ*, TBET, IL-4, C–C motif chemokine ligand 12 (CCL12), and CCL22 [36]. In addition, treatment with praziquantel at a dose of 80 mg/kg 50 days postinfection for 5 consecutive days was reported to result in a significant reduction in the volume density of hepatocytes, sinusoids, and hepatic fibrosis in mice infected with *S. mansoni* and fed either a low-protein diet (8% protein) or standard chow (22% protein) [37]. In a recent study, however, treatment with praziquantel at a dose of 400 mg/kg twice daily 12 weeks postinfection for a week followed by praziquantel treatment at a dose of 400 mg/kg twice daily for 4 weeks was found to achieve comparable collagen deposition, hydroxyproline level, and granuloma areas relative to untreated controls in the murine model of chronic experimental schistosomiasis mansoni [38] (Table 1).

In addition, combined therapy of praziquantel with alpha lipoic acid [39], silymarin [40], AT1 receptor antagonist losartan [41], was reported to achieve a greater activity against hepatic fibrosis induced by *S. mansoni* infection than praziquantel monotherapy. Since stem cell therapy has shown a potential value in treatment of schistosomiasis-associated fibrosis [42–44], the antifibrotic activity of human Wharton's jelly-derived mesenchymal stem cells in combination with praziquantel was evaluated in *S. mansoni*-infected mice, and the combination was found to achieve a higher beneficial efficacy against *S. mansoni*-induced liver fibrosis than monotherapy with mesenchymal stem cells or praziquantel alone, as revealed by histopathological, morphometric, and gelatin zymographic results as well as reduction of alpha smooth muscle actin (*α*-SMA), Col1, and IL-13 expression [45].

In animal models of fibrosis induced by *S. japonicum*, praziquantel treatment was found to reduce serum alanine aminotransferase (ALT) and aspartate aminotransferase (AST) concentrations, the areas of egg granulomas, areas of collagen deposition and hepatic hydroxyproline contents, alleviate fibrotic proliferation, inflammatory infiltration, hepatocyte degeneration and necrosis, improve liver size and texture, decrease the levels of fibrosis-related parameters including *α*-SMA, SPET4, matrix metalloproteinase (MMP)-9, tissue inhibitor of metalloproteinase (TIMP)-1, Col1*α*1, and Col3*α*1 in relative to infected but untreated controls, and inhibit the expression of proinflammatory cytokines tumor necrosis factor (TNF)-*α* and TGF-*β*1[31, 46–52] (Table 2). Combined treatment with praziquantel and IFN-*γ* was reported to result in a greater decline in hepatic fibrosis score and area of collagen deposition, higher reduction of Col1 and Col3 expression, more downregulation of Smad2 expression and upregulation of Smad7 expression as compared to treatment with praziquantel alone [53, 54], and the combination of praziquantel and the *Amusium pleuronectes* polysaccharide presented a more inhibitory effect on hepatic fibrosis than treatment with praziquantel or the polysaccharide alone in *S. japonicum*-infected mice, as revealed by lower Col1, Col2, and Col4 levels, lower parasite egg burdens and higher IL-12 and IFN-*γ* productions [55]. In addition, combining praziquantel and extracts from traditional Chinese medicines achieved synergistic activity in the treatment of *S. japonicum*-induced hepatic fibrosis [56–58].

The results of these experimental studies demonstrate the effectiveness of praziquantel against hepatic fibrosis induced by *S. mansoni* or *S. japonicum* infection, which encourages the randomized, controlled clinical trials to confirm the antifibrotic activity of praziquantel.

### 2.2. Exciting Findings from Human Trials

To date, there are no randomized, controlled trials to examine the role of praziquantel in prevention and control of fibrosis associated with schistosomiasis; however, available data have shown the antifibrotic value of praziquantel in treatment of patients with schistosomiasis-induced fibrosis [30, 59]. In *S. mansoni*-infected subjects, administration of praziquantel at a total dose of 40 or 50 mg/kg resulted in improvements in the ultrasonographic parameters of fibrosis [60–64] (Table 3). Among *S. japonicum*-infected patients, praziquantel treatment at a total dose of 40 or 60 mg/kg was found to improve the ultrasonographic and biochemical parameters in patients with mild fibrosis but not in patients with severe fibrosis [30, 65–68] (Table 4); however, Fabre and colleagues reported an elevated prevalence of hepatic fibrosis in *S. japonicum*-infected Filipinos following one-year treatment of praziquantel at a total dose of 60 mg/kg given in a split dose [69]. Furthermore, multicenter, randomized, double-blind, and controlled clinical trials are required to validate the antifibrotic efficacy of praziquantel.

### 2.3. Mechanisms Underlying Antifibrotic Activity of Praziquantel against Schistosoiasis-Induced Fibrosis

Previous studies have demonstrated that praziquantel is active against schistosomiasis-induced hepatic fibrosis through decreasing the expression of fibrotic biomarkers including *α*-SMA, collagen, MMP, and TIMP, and inhibiting the expression of proinflammatory cytokines such as IL-6, TNF-*α,* and TGF-*β*, as well as chemokines [31–34, 47–52]. In addition, praziquantel treatment resulted in a high proportion of the active form of the interstitial collagenase [70], inhibition of SEPT4 expression at both translational and transcriptional levels [48], and increased plasminogen activator activity [71], which was considered to contribute to the reversal of schistosomiasis-induced fibrosis. Since multiple factors are involved in schistosomiasis-induced fibrosis, the exact mechanisms underlying the action of praziquantel against schistosomiasis-induced fibrosis require further investigations.

## 3. Conclusions and Perspectives

Currently, there are no effective treatments for hepatic fibrosis except liver transplantation [19]. Early diagnosis and interventions are of great importance to attenuate hepatic fibrosis; however, there are still challenges for early diagnosis of hepatic fibrosis [72]. Nevertheless, artificial intelligence (AI) opens the new era of precision medicine in hepatology, which facilitates early precise identification and prediction of disease severity and progression, the presence of complications, and outcomes of hepatic fibrosis [73–75]. In addition, stem cell therapy has shown great potential in the treatment of hepatic fibrosis, which provides a new hope for the treatment of hepatic fibrosis [76–78]; however, further prospective clinical trials to examine the efficacy and safety of stem cell therapy for hepatic fibrosis associated with schistosomiasis are required.

It has been proven that praziquantel is a highly effective and mildly toxic schistosomicide [79], and praziquantel-based chemotherapy has been widely implemented in the national schistosomiasis control program around the world and plays a great role in the transmission of schistosomiasis [80]. In addition to antischistosomal action, both experimental studies and clinical trials have revealed the antifibrotic activity of praziquantel against hepatic fibrosis induced by *S. japonicum* and *S. mansoni* infection [47–52,61–68]. Moreover, oral administration of praziquantel at a dose of 300 mg/kg given twice daily for 30 days resulted in a significant reduction of the collagen areas, content of hepatic hydroxyproline, and serum ALT and AST levels in a CCl_4_-induced mouse model of hepatic fibrosis [30]. In the concanavalin A (Con A)-induced model of hepatic fibrosis, praziquantel was also found to improve the morphological and biochemical parameters associated with hepatic fibrosis [81]. Since hepatic fibrosis may be induced by virus, alcohol and toxin [82–84], and there are millions of subjects suffering from hepatic fibrosis due to viral hepatitis, parasitic diseases, toxic chemicals, and alcohol consumption, further studies to investigate the potential of praziquantel against alcoholic, viral, and toxin-induced fibrosis seem justified.

In summary, praziquantel, an old schistosomicide, is a promising antifibrotic drug. Although randomized, controlled clinical trials are required to validate the antifibrotic action, and there are still a large number of challenges ahead, the antifibrotic activity of praziquantel may benefit thousands of patients, due to its low cost, easy administration, and low toxicity. In addition, the timing, dosing, optimal regimens, and mechanisms of action of praziquantel for treatment of fibrosis require further investigation.

## Figures and Tables

**Table 1:** Activity of praziquantel monotherapy against hepatic and splenic fibrosis associated with schistosomiasis mansoni.

Animal model	Pattern of fibrosis	Treatment regimen	Effect on schistosomiasis-induced fibrosis	Reference
Mouse	Hepatic fibrosis	Administration of praziquantel at a dose of 250 mg/kg 8 weeks postinfection	A prompt reduction in liver parasite egg load with no viable eggs, and a moderate decrease in liver fibrosis 10 weeks post-treatment; and arrest of fibrosis and reduction of liver collagen content to normal levels by 20 weeks post-treatment	32

Swiss albino mouse	Hepatic fibrosis	Administration of praziquantel at a dose of 250 mg/kg 8, 12, and 20 weeks postinfection	A reduction of total collagen content and recovery of type III/I collagen ratio to normal limits following treatment given 8 weeks postinfection	33

Syrian golden hamster	Hepatic fibrosis and splenic fibrosis	Administration of praziquantel at a dose of 100 mg/kg 12, 13, 14, and 15 weeks postinfection	A significant reduction of granulomas, CAA and CCA in hepatic specimens and a clear-cut reduction of fibrosis, granulomas, CAA and CCA in splenic specimens	34

CBA/J^k^ mouse	Hepatic fibrosis	Administration of praziquantel at a dose of 300 mg/kg for 2 days 8 weeks postinfection	Amelioration of hepatic granulomatous pathology, reduction of collagen I, III, and IV gene expression at 6 and 12 months post-treatment, and resorption of liver fibrous tissue by 71.4% 12 months post-treatment	35

Swiss webster mice	Hepatic fibrosis	Administration of praziquantel at a dose of 250 mg/kg 32 days postinfection for 4 consecutive days	Elimination of egg granulomas or collagen fibrils, and a reduction in the expression of STAT1, STAT4, IFN-*γ*, TBET, IL-4, CCL12, and CCL22	36

Swiss webster mice	Hepatic fibrosis	Treatment with praziquantel at a dose of 80 mg/kg 50 days postinfection for 5 consecutive days	A significant reduction in the volume density of hepatocytes, sinusoids, and hepatic fibrosis	37

Balb/C mouse	Hepatic fibrosis	Treatment with praziquantel at a dose of 400 mg/kg twice daily 12 weeks postinfection for a week followed by praziquantel treatment at a dose of 400 mg/kg twice daily for 4 weeks	No significant reduction in collagen deposition, hydroxyproline level, or granuloma areas	38

CAA, circulating anodic antigen; CCA, circulating cathodic antigen; STAT, signal transducer and activator of transcription; IFN, interferon, IL, interleukin; CCL, C–C motif chemokine ligand.

**Table 2:** Activity of praziquantel monotherapy against hepatic and splenic fibrosis associated with schistosomiasis japonica.

Animal model	Pattern of fibrosis	Treatment regimen	Effect on schistosomal fibrosis	Reference
BABL/*c* mouse	Hepatic fibrosis	Praziquantel at a dose of 300 mg/kg twice daily for 30 days administered 8 and 15 weeks postinfection	A significant reduction in the areas of sirius red-stained liver, liver hydroxyproline contents, spleen weight, spleen index, and levels of Col1*α*1, Col3*α*1, *α*-SMA, MMP9, and TIMP1 as compared to infected but untreated controls	31

Rabbit	Hepatic fibrosis	Oral administration of praziquantel at a single dose of 100 mg/kg 6, 12 or 24 weeks postinfection	A significant decrease in portal vein pressure, number and size of egg granulomas, and liver collagen content, and improvement of echogenic bands and nodules	46

Kunming mouse	Hepatic fibrosis	Administration of praziquantel at a daily dose of 500 mg/kg 8 weeks postinfection for 2 days, followed by praziquantel treatment twice a week for 8 weeks	Significantly reduced egg granulomas area, type I and type III collagen levels, and ALT and AST activities, remarkable improvements in liver size and texture, and significantly reduced hepatic fibrosis degree as compared to infected but untreated controls	47

ICR mouse	Hepatic fibrosis	Praziquantel given at a daily dose of 250 mg/kg for 3 days 6 weeks postinfection	A clear-cut decline in diameters of egg granulomas, areas of collagen deposition and *α*-SMA expression, inhibition of TNF*-α* and IL-6 mRNA expression, and reduced SEPT4 expression at transcriptional and translation levels as compared to infected but untreated controls	48

Kunming mouse	Hepatic fibrosis	Administration of praziquantel at a daily dose of 500 mg/kg 2 weeks postinfection for 2 days, followed by praziquantel treatment twice a week for 8 weeks	Alleviated fibrotic proliferation and inflammatory infiltration, significantly reduced egg granulomas and hepatocyte degeneration and necrosis, and significantly decreased serum NO, hepatic inducible nitric oxide synthase (iNOS), TGF-*β*1, type I and type III collagen, and TNF-*α* levels, and significantly elevated Bcl-2 and INF-*γ* levels as compared to infected but untreated controls	49–52

Col1*α*1, collagen I*α*1; Col3*α*1, collagen III*α*1; *α*-SMA, *α*-smooth muscle actin; MMP9, matrix metalloproteinase-9; TIMP1, metalloproteinase-1; TNF-*α*, tumor necrosis factor *α*; TGF-*β*1, transforming growth factor *β*1; IL-6, interleukin-6; INF-*γ*, interferon-*γ*; ALT, alanine transaminase; AST, aspartate transaminase.

**Table 3:** Clinical efficacy of praziquantel in treatment of patients with schistosomiasis mansoni-induced fibrosis.

Pattern of fibrosis	Treatment regimen	Clinical outcomes of fibrosis	Reference
Hepatic periportal fibrosis	Praziquantel at a total dose of 40 mg/kg	Improvement of periportal thickening/fibrosis in 17.6% of the cohorts, total resolution in 34.7% 26-months post-treatment	60
Hepatic periportal fibrosis	Praziquantel at a total dose of 40 mg/kg	Reduction of hepatic periportal thickening from 46% at baseline to 32% one-year post-treatment and 35% three-year post-treatment	61
Hepatic periportal fibrosis	Praziquantel at a total dose of 50 mg/kg	Significant reduction in the mean values for longitudinal and anteroposterior measurements of liver (left and right lobes) as well as portal vein diameter, a considerable reduction in moderate fibrosis and IL-10 level one-year post-treatment	62
Hepatic periportal fibrosis	Praziquantel at a total dose of 40 mg/kg	Complete reversal of periportal lesions seen in 28.2% subjects; a reduction in periportal thickening followed by a decrease in the size of the left hepatic lobe 3 to 5 years post-treatment	63
Hepatic periportal fibrosis	Praziquantel at a total dose of 40 mg/kg	Improvement in the sonomorphological abnormalities of periportal fibrosis and organomorphometry of livers and spleens one-year post-treatment	64

**Table 4:** Clinical efficacy of praziquantel in treatment of patients with schistosomiasis japonica-induced fibrosis.

Pattern of fibrosis	Treatment regimen	Clinical outcomes of fibrosis	Reference
Hepatic fibrosis	Praziquantel at a total dose of 60 mg/kg	Decreased length of the left liver lobe, the spleen, the ratio of the exterior diameter to the interior diameter of the second branch of the portal vein; significantly reduced abnormal rates of serum hyaluronic acid, and type III procollagen levels one-year post-treatment	30
Hepatic fibrosis	Praziquantel at a single dose of 40 mg/kg	Significant improvement of ultrasound parenchyma images and periportal fibrosis in 37% and 54% of the cohort, and reversal of left-lobe liver enlargement two-year post-treatment	65
Hepatic fibrosis	Praziquantel given at 3 doses of 20 mg/kg	Improvement in the thickening of the portal vein wall and the intensity of echogenic bands; significant decrease in serum total bile acid level; but no significant ultrasonographic changes seen in patients with severe hepatic fibrosis 6 months post-treatment	66
Hepatic fibrosis	Praziquantel given at 3 doses of 20 mg/kg	Significant improvement in mild hepatic fibrosis, which was not seen in patients with severe fibrosis; no changes of the network pattern of the echogenic bands; no change of serum total bile acid level one-year post-treatment	67
Hepatic fibrosis	Praziquantel given at a dose of 60 mg/kg divided in two days	Significant improvements of clinical symptoms (abdominal distension, rib pain, diarrhea, and weakness) in 90% participants, shrinkage of liver and spleen size in 80% participants, complete recovery of serum hyaluronic acid, laminin, type IV collagen, and type III procollagen levels to normal in 64 participants three years post-treatment	68

## Data Availability

All data presented in this study are available from the corresponding author upon request.
